# nPTD classification: an updated classification of gastric cancer location for function preserving gastrectomy based on physiological lymphatic flow

**DOI:** 10.1186/s12885-021-08936-9

**Published:** 2021-11-17

**Authors:** Shinichi Kinami, Naohiko Nakamura, Tomoharu Miyashita, Hidekazu Kitakata, Sachio Fushida, Takashi Fujimura, Tohru Itoh, Hiroyuki Takamura

**Affiliations:** 1 Department of Surgical Oncology, Kanazawa Medical University, 1-1 Daigaku, Uchinada-machi, Kahoku-gun, Ishikawa 920-0293 Japan; 2 Department of Gastroenterological Endoscopy, Kanazawa Medical University, Kahoku-gun, Ishikawa Japan; 3 Department of Gastroenterologic Surgery, Kanazawa University, Kanazawa, Ishikawa Japan; 4 Department of Surgery, Toyama City Hospital, Toyama, Japan

**Keywords:** Gastric cancer, Sentinel node biopsy, Lymphatic flow, Function preserving gastrectomy

## Abstract

**Background:**

The correlation between tumor location and lymphatic flow distribution in gastric cancer has been previously reported, and PTD (Proximal – Transitional – Distal) classification was proposed. Our group updated and developed the nPTD classification.

**Method:**

We retrospectively studied gastric cancer patients who underwent the dye method sentinel node biopsy from 1993 to 2020. The inclusion criteria were a single lesion type 0 cancer of ≤5 cm in the long axis, clinically node-negative, and invasion within the proper muscle layer pathologically. In this study, the distribution of dyed lymphatic flow was evaluated for each occupied area of the tumor.

**Results:**

We included 416 patients in this study. The tumors located in the watershed of the right and left gastroepiploic arteries near greater curvature had extensive lymphatic flow; therefore, a newly circular region with a diameter of 5 cm is set on the watershed of the greater curvature between P and T zone as the ‘n’ zone. In addition, for cancers located in the lesser P curvature, lymphatic flow to the greater curvature was not observed. Therefore, the P zone was divided into two: the lesser curvature side (PL) and the greater curvature side (PG).

**Conclusions:**

The advantage of the nPTD classification is that it provides not only proper nodal dissection but also adequate function-preserving gastrectomy. If the tumor is localized within the PL, the proximal gastrectomy resection area can be further reduced. In contrast, for cancers located in the ‘n’ zone, near-total gastrectomy is required because of the extensive lymphatic flow.

## Background

Gastric cancer location is generally described based on the Japanese classification of gastric carcinoma [1, 2]. The longitudinal axis is divided into three areas: upper (U), middle (M), and lower (L). This is simply divided into three equal parts: the lesser curvature and greater curvature of the stomach. However, the most important aspect of gastric cancer curative surgery is the lymph node dissection [3, 4], and for surgical treatment, classifying tumor location using lymphatic flow is useful.

**Fig. 1:**
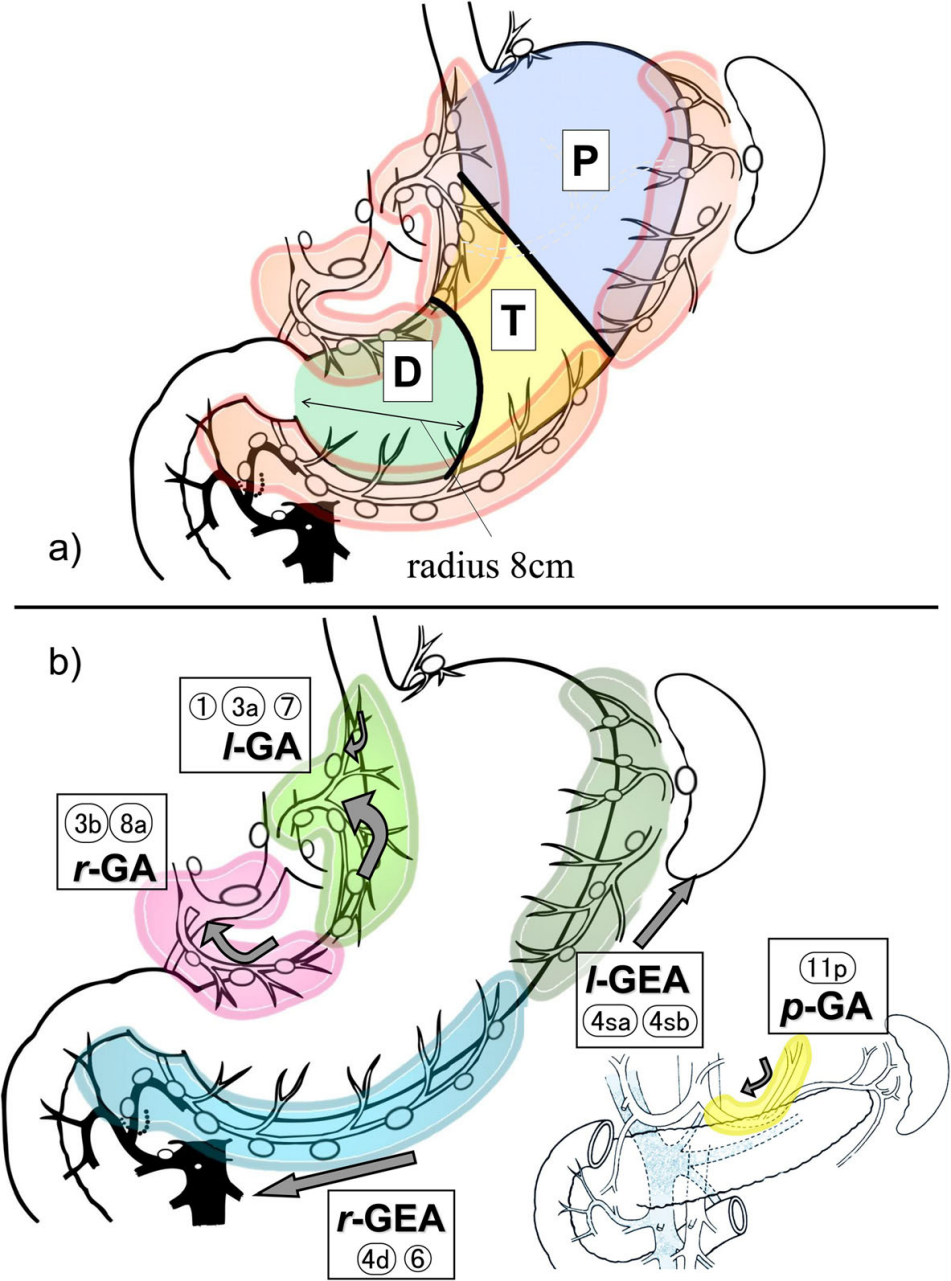
The PTD classification and the lymphatic compartment for gastric cancer. **a** PTD classification. The proximal region is named zone P, the distal region is named zone D, and the transitional region is named zone T. **b** The gastric lymphatic compartments of the stomach. Lymphatic basins were found within these five compartments. *l*-GA, left gastric artery basin; *r*-GA, right gastric artery basin; *r*-GEA, right gastroepiploic artery basin; *l*-GEA, left gastroepiploic artery basin; *p*-GA, posterior gastric artery basin. These figures were newly created by modifying the figures in the study by Kinami S et al.: Int J Clin Oncol. 2008;13:320–9 [5].

Previously, our group found a correlation between gastric cancer tumor location and lymphatic flow distribution and proposed the Proximal–Transitional–Distal (PTD) classification based on gastric cancer location and lymphatic flow (Fig. 1a) [5]. The PTD classification is derived from gastric cancer-specific lymphatic flow observation using the dye method of sentinel node biopsy. Presently, sentinel node biopsy is the most reliable diagnostic method to identify lymph node metastasis in early gastric cancer [6]. The PTD classification is better than UML classification because it provides proper lymph node dissection index and guides function-preserving gastrectomy for early gastric cancer, such as proximal and segmental gastrectomies [5, 7].

Subsequently, sentinel node biopsy is continued, and further lymphatic flow regularity is confirmed in early gastric cancer. Thus, the PTD classification was updated, and the nPTD classification, a more suitable clinical practice tool, was developed.

## Methods

This is a retrospective study of gastric cancer patients who underwent sentinel node biopsy both at Kanazawa University Hospital from 1993 to 2008 and at Kanazawa Medical University Hospital from 2009 to 2020. The sentinel lymph node biopsy indication for gastric cancer is the presence of a type 0 single lesion gastric cancer ≤5 cm in the long axis, which is clinically node-negative (cN0), as diagnosed by preoperative computed tomography [5, 8, 9]. In this study, patients included were those with an observable lymphatic flow during sentinel node mapping, using the dye method, while patients with > 5 cm in the long axis or a pathologically invaded layer deeper than the subserosal layer, and those whose accurate lymphatic flow was not observed were excluded.

At Kanazawa University Hospital, mapping was performed using the blue dye or the radioisotope (RI) and dye combination methods, and at Kanazawa Medical University Hospital, mapping was performed mainly by using the indocyanine green (ICG) fluorescence method. In the blue dye or the RI and dye combination methods, 0.2 ml of 2% sulphan blue was injected endoscopically into the submucosal layer’s four points around the tumor using the endoscopic injection needle immediately before or during surgery. The lymphatic flow was observed after 15 min, and the lymphatic basin and sentinel nodes were detected and recorded. Lymph nodes stained blue were regarded as sentinel lymph nodes [8]. In the ICG fluorescence method, ICG diluted to 50 μg/mL was used as a tracer; 0.5 mL of the tracer was injected endoscopically into the submucosal layer’s four points around the tumor for mapping the day before surgery. A small amount of ICG administered was sensitized and detected using Photodynamic Eye (PDE Hamamatsu Photonics). The lymphatic basin (basin through which the ICG fluorescent lymphatic vessel flow) was identified and recorded. Lymph nodes with clear fluorescence when observed with PDE were regarded as sentinel nodes [9].

The lymphatic basin is a certain lymphatic area that drains directly from the primary focus and is specific for gastric cancer. According to the previous report, lymphatic basins were defined as follows: the proximal side was the stomach wall, and the distal side was the most distal dye-stained lymph node [5]. Sentinel nodes are found only within lymphatic basins, which are integrated into the five lymphatic areas shown in Fig. 1b, except for the lymphatic flow to the left paracardial lymph node (No. 2 lymph node, #2). As previously reported, each of these is called the lymphatic compartment and is classified into five basins: the left gastric artery basin (*l*-GA), right gastric artery basin (*r*-GA), left gastroepiploic artery basin (*l*-GEA), right gastroepiploic artery basin (*r*-GEA), and the posterior gastric artery basin (*p*-GA). Classifying the lymphatic flow to #2 is challenging because of the multidirectional flow to *l*-GA and No. 19 lymph node ahead and the lymphatic flow to *p*-GA nearby. Therefore, it was excluded from the lymphatic compartment classification and handled separately [5, 8].

The lymphatic basin distribution was tabulated for each gastric cancer location, and gastric lymphatic flow regularity was examined. The UML was classified according to the Japanese gastric carcinoma classification [1]. Since it is clinically difficult to determine the boundary between U and M, the lesions located at the boundary between U and M (UM and MU) were counted separately in this study. The PTD classification was based on a previous report [5]. The boundary between zones P and T is the line connecting the point of the watershed between the left and right gastroepiploic arteries and the inflow point of the first descending branch of the left gastric artery. An arc with a radius of 8 cm from the pylorus was defined as the boundary between zones T and D. All tumor regions were determined preoperatively based on UML classification. According to the PTD classification, tumor regions were determined retrospectively from the endoscopic findings and surgical records of cases from Kanazawa University Hospital and preoperatively for cases from Kanazawa Medical University Hospital. Furthermore, we attempted to improve the PTD classification based on the distribution of lymphatic flow. Gastric cancer has been described following the Japanese classification of gastric carcinoma [1].

The chi-square test was used to compare background factors. *P*-values < 0.05 were considered significant. All statistical analyses were performed using EZR (Saitama Medical Center, Jichi Medical University, Saitama, Japan), a graphical user interface of R (The R Foundation for Statistical Computing, Vienna, Austria). EZR is a modified version of R Commander [10].

This study was approved by the ethics committee of Kanazawa University Hospital and Kanazawa Medical University (Trial Number R093 (28/08/2009), M288 (25/02/2013)) and registered with the University Hospital Medical Information Network’s Clinical Trials Registry (trial number UMIN000010154 and UMIN000023828). ICG mapping was approved by the ethics committee of Kanazawa Medical University (Trial Number M404 (25/07/2016) and jRCTs041180006 https://jrct.niph.go.jp/latest-detail/jRCTs041180006).

This study was conducted following the Good Clinical Practice guidelines and the Declaration of Helsinki. All patients provided written informed consent for surgery and use of their data. Regarding data used for lymph node mapping, patients were allowed to opt out of the study at any time.

## Results

### The diagnostic ability of sentinel node biopsy

Overall, 484 patients underwent gastric cancer sentinel node biopsies between 1993 and 2020. However, 416 met the inclusion criteria. The patients’ characteristics are shown in Table 1. The total number of occupied areas was U 54, UM/MU 13, M 215, and L 134. The occupying area distribution of blue dye and ICG fluorescence groups showed a slight difference (U cancers were slightly more common in the ICG group). However, no difference was observed in the other parameters. There were 42 cases of nodal metastases. Sentinel node biopsy diagnostic sensitivity was 85.7% (36/42) with 98.6% (410/416) accuracy. Of the 36 metastasis-positive patients, 20 (56%) had only sentinel node metastasis. Six false-negative cases were observed, of which three had macroscopic metastatic nodes, with the tracer not entering these obvious metastases, while the other three were false negatives for rapid intraoperative frozen-section diagnosis. The former patients were easily diagnosed as metastatic cases without a sentinel node biopsy. The latter encountered an unavoidable problem in the intraoperative pathological diagnosis; that is, no metastases were found on the frozen section, but they were found in the permanent sections that were re-cut after formalin fixation. Therefore, we did not encounter any true false-negative cases. Regarding survival prognosis, there was no gastric cancer recurrence in the 374 metastasis-negative cases. One of the false-negative cases of rapid intraoperative diagnosis died of pancreatic cancer, but two were alive without recurrence. The other 39 metastasis-positive patients underwent standard nodal dissection, of which five had a gastric cancer recurrence.

**Table 1:** Patients’ characteristics

Number of patients	Total	Blue dye	ICG	*p*-value
	416	323	93	n.s.
Age (year)				
median (range)	64 (26–86)	62 (26–86)	70 (42–84)	n.s.
Sex				
Male / Female	278 / 138	220 / 103	58 / 35	n.s.
Location of tumor				
U / UM·MU / M / L	54/13/215/134	36/7/171/109	18/6/44/25	0.026
Size of tumor				
mm (long axis)	22 (2–50)	25 (2–50)	25 (8–50)	n.s.
Macroscopic type				
elevated / depressed	97 / 319	77 / 246	20 / 73	n.s.
Pathological depth				
T1a(M) / T1b(SM) / T2(MP)	210 / 166 / 40	171 / 121 / 31	39 / 45 / 9	n.s.
Pathological type				
differentiated / undifferentiated	243 / 173	184 / 139	59 / 34	n.s.
Surgical procedures				
standard / modified	140 / 276	108 / 215	32 / 61	n.s.
Nodal metastatic status				
SN(+) nonSN(−)	20	13	7	
SN(+) nonSN(+)	16	11	5	
SN(−) nonSN(+)	6	5	1	
SN(−) nonSN(−)	374	294	80	

Statistical tests were performed between the blue dye and ICG groups

*SN* sentinel node

### The distribution pattern of the lymphatic basins

Table 2 shows the number and distribution of the observed lymphatic compartments by the UML classification. The most common lymphatic compartment was *l*-GA, found in 83.2% of patients, followed by *the r*-GEA. No lymphatic flow to *r*-GA and *r*-GEA was found in cancers confined to the U-region. In addition, lymphatic flow to *p*-GA and # 2 was observed in U cancers only. Lymphatic flow to *l*-GEA was not observed in L cancers, whereas it was observed in 4.2% of M cancers and 46.2% of UM/MU cancers. The number and distribution of lymphatic basins differed in each case depending on the tumor’s location, with a minimum of one basin and a maximum of three basins. 10% of patients with U or M cancers were found to have three basins, but this value exceeded 30% in those with UM/MU or L cancers. The distribution patterns by blue dye and ICG are shown in Table 3. The basin distribution between the blue dye and the ICG fluorescence groups showed no significant difference. We also examined whether the tumor size, macroscopic type, pathological depth, and pathological type affected the distribution pattern of basins; however, we found that these factors did not affect the distribution patterns.

**Table 2:** The number and distribution of observed lymphatic compartments by UML and PTD classifications

Tumor location	All site	U	UM·MU	M	L	P	T	D
No. of patients	416	54	13	215	134	57	176	183
Distribution of basin								
*l*-GA	83.2%	98.1%	92.3%	89.8%	65.7%	96.5%	90.9%	71.6%
*r*-GA	27.9%	**0.0%**	**0.0%**	17.7%	58.2%	**0.0%**	**0.0%**	63.4%
*r*-GEA	63.0%	**0.0%**	46.2%	66.0%	85.1%	**3.5%**	68.8%	76.0%
*l*-GEA	5.5%	14.8%	46.2%	**4.2%**	0.0%	26.3%	**4.5%**	0.0%
*p*-GA	1.4%	11.1%	0.0%	0.0%	0.0%	10.5%	0.0%	0.0%
#2	3.1%	24.1%	0.0%	0.0%	0.0%	22.8%	0.0%	0.0%
Number of basins								
1	33.9%	61.1%	46.2%	32.6%	23.9%	52.6%	39.8%	22.4%
2	48.3%	29.6%	23.1%	56.3%	45.5%	35.1%	56.3%	44.8%
3	17.8%	9.3%	30.8%	11.2%	30.6%	12.3%	4.0%	32.8%

All percentage numbers represent the percentage of confirmed lymphatic flow per tumor location

*#2* No. 2 lymph node, *l-GA* left gastric artery basin, *l-GEA* left gastroepiploic artery basin, *p-GA* posterior gastric artery basin, *r-GA* right gastric artery basin, *r-GEA* right gastroepiploic artery basin

**Table 3:** Number and distribution of observed lymphatic compartments by UML and PTD classifications for each mapping procedure

a. Blue dye mapping								
Tumor location	All site	U	UM·MU	M	L	P	T	D
No. of patients	323	36	7	171	109	40	141	142
Distribution of basins								
*l*-GA	81.4%	97.2%	85.7%	88.9%	64.2%	95.0%	90.8%	68.3%
*r*-GA	28.2%	0.0%	0.0%	17.5%	56.0%	0.0%	0.0%	64.1%
*r*-GEA	64.4%	0.0%	28.6%	64.9%	87.2%	0.0%	68.1%	78.9%
*l*-GEA	5.9%	19.4%	42.9%	5.3%	0.0%	30.0%	5.0%	0.0%
*p*-GA	1.5%	13.9%	0.0%	0.0%	0.0%	12.5%	0.0%	0.0%
#2	2.5%	22.2%	0.0%	0.0%	0.0%	20.0%	0.0%	0.0%
Number of basins								
1	34.1%	58.3%	57.1%	33.9%	24.8%	52.5%	40.4%	22.5%
2	48.3%	30.6%	28.6%	54.4%	45.9%	37.5%	55.3%	44.4%
3	17.6%	11.1%	14.3%	11.7%	29.4%	10.0%	4.3%	33.1%
b. ICG fluorescence mapping								
Tumor location	All site	U	UM·MU	M	L	P	T	D
No. of patients	93	18	6	44	25	17	35	41
Distribution of basins								
*l*-GA	89.2%	100.0%	100.0%	93.2%	72.0%	100.0%	91.4%	82.9%
*r*-GA	26.9%	0.0%	0.0%	18.2%	68.0%	0.0%	0.0%	61.0%
*r*-GEA	58.1%	0.0%	66.7%	70.5%	76.0%	11.8%	71.4%	65.9%
*l*-GEA	4.3%	5.6%	50.0%	0.0%	0.0%	17.6%	2.9%	0.0%
*p*-GA	1.1%	5.6%	0.0%	0.0%	0.0%	5.9%	0.0%	0.0%
#2	5.4%	27.8%	0.0%	0.0%	0.0%	29.4%	0.0%	0.0%
Number of basins								
1	33.3%	66.7%	33.3%	27.3%	20.0%	52.9%	37.1%	22.0%
2	48.4%	27.8%	16.7%	63.6%	44.0%	29.4%	60.0%	46.3%
3	18.3%	5.6%	50.0%	9.1%	36.0%	17.6%	2.9%	31.7%

All percentage numbers represent the percentage of confirmed lymphatic flow per tumor location

*#2* No. 2 lymph node, *l-GA* left gastric artery basin, *l-GEA* left gastroepiploic artery basin, *p-GA* posterior gastric artery basin, *r-GA* right gastric artery basin, *r-GEA* right gastroepiploic artery basin

Using the PTD classification for the distribution of lymphatic compartments (Table 2), the lymphatic flow to *r*-GA was not observed in P or T cancers, while lymphatic flow to *p*-GA or # 2 was not observed in T or D cancers. In P cancers, the lymphatic flow was 26, 10, and 23% to *l*-GEA, *p*-GA, and # 2, respectively. Lymphatic flow to *r*-GEA was observed in two P cancers patients (3.5%). One was UM Gre 30 × 25 mm 0 IIc + III pT1b2 (SM2) por2, and the other was MU Gre 25 × 15 mm 0 IIc pT1a (M) por2. Both cases were cancers on the greater curvature of the UM·MU region.

In addition, lymphatic flow to *l*-GEA was observed in eight patients (4.5%) with T cancers. (Table 4). The tumors were located at the greater curvature or posterior wall. They were often poorly differentiated adenocarcinomas; most had an extensive lymphatic flow to *l*-GA, *l*-GEA, *r*-GEA, and were close to the right and left gastroepiploic arteries watershed.

**Table 4:** An overview of the eight patients located in the T zone who had a lymphatic flow to *l*-GEA

Mapping	UML	Circum.	Size (mm)	M type	Patho.	Depth	Lymphatic basins
Blue dye	UM	GreAnt	50	IIc	por2	sm2	*l*-GA *r*-GEA *l*-GEA
Blue dye	M	GrePost	40	IIa + IIc	por1	mp	*l*-GA *r*-GEA *l*-GEA
Blue dye	M	GrePost	35	IIc	por2	m	*l*-GA *r*-GEA *l*-GEA
Blue dye	M	GrePost	32	I	tub1	sm2	*l*-GA *l*-GEA
Blue dye	M	PostGre	13	IIc	sig	m	*l*-GA *r*-GEA *l*-GEA
Blue dye	M	Gre	12	IIc	sig	m	*l*-GA *r*-GEA *l*-GEA
Blue dye	M	GrePost	12	IIc	tub1	m	*l*-GA *r*-GEA *l*-GEA
ICG	UM	Post	10	IIc	tub2	sm1	*l*-GA *r*-GEA *l*-GEA

*Circum.* circumferential location of the tumor, *Depth* depth of invasion, *ICG* ICG fluorescence method, *Patho* pathological type due to Japanese Classification of Gastric cancer, *Size* the size of the long axis of the tumor

### Lymphatic flow to the contralateral side

Cancers in the lesser curvature or greater curvature were extracted and are summarized in Table 5. Lymphatic flow to the lesser curvature was observed at a high rate in the greater curvature, regardless of the occupying longitudinal region. Conversely, among cancers with the lesser curvature, 27% of T cancers and 59% of D cancers were lymphatic flow at the greater curvature, while P cancers did not.

**Table 5:** Lymphatic flow to the contralateral side distribution of observed lymphatic compartments in the lesser or greater curvature

Circumference	Lesser curvature			Greater curvature		
Location of tumor	P	T	D	P	T	D
Number of patients	29	59	96	5	47	30
*l*-GA	100.0%	98.3%	83.3%	**100.0%**	**68.1%**	**36.7%**
*r*-GA	0.0%	0.0%	71.9%	0.0%	0.0%	**43.3%**
*r*-GEA	0.0%	**27.1%**	**59.4%**	40.0%	97.9%	100.0%
*l*-GEA	**0.0%**	0.0%	0.0%	80.0%	6.4%	0.0%
*p*-GA	10.3%	0.0%	0.0%	0.0%	0.0%	0.0%
#2	17.2%	0.0%	0.0%	20.0%	0.0%	0.0%

All percentage numbers represent the percentage of confirmed lymphatic flow per tumor location

*#2* No. 2 lymph node, *l-GA* left gastric artery basin, *l-GEA* left gastroepiploic artery basin, *p-GA* posterior gastric artery basin, *r-GA* right gastric artery basin, *r-GEA* right gastroepiploic artery basin

### Update of PTD classification

PTD classification was updated based on this result. Even if the number of cases increased, there were no cases in which lymphatic flow to *r*-GA was observed in T cancer, and the boundary between T and D did not need to be changed. On the other hand, there were P cancer patients with the lymphatic flow to r-GEA and T cancer to *l*-GEA. These cancers were located in the greater curvature near the watershed of the right and left gastroepiploic arteries and had an extensive lymphatic flow to *l*-GA, *l*-GEA, and *r*-GEA. In addition, lymphatic flow to the contralateral side was observed mostly in T and D cancers; however, lymphatic flow to the greater curvature was not observed in cancers in the lesser P curvature.

**Fig. 2:**
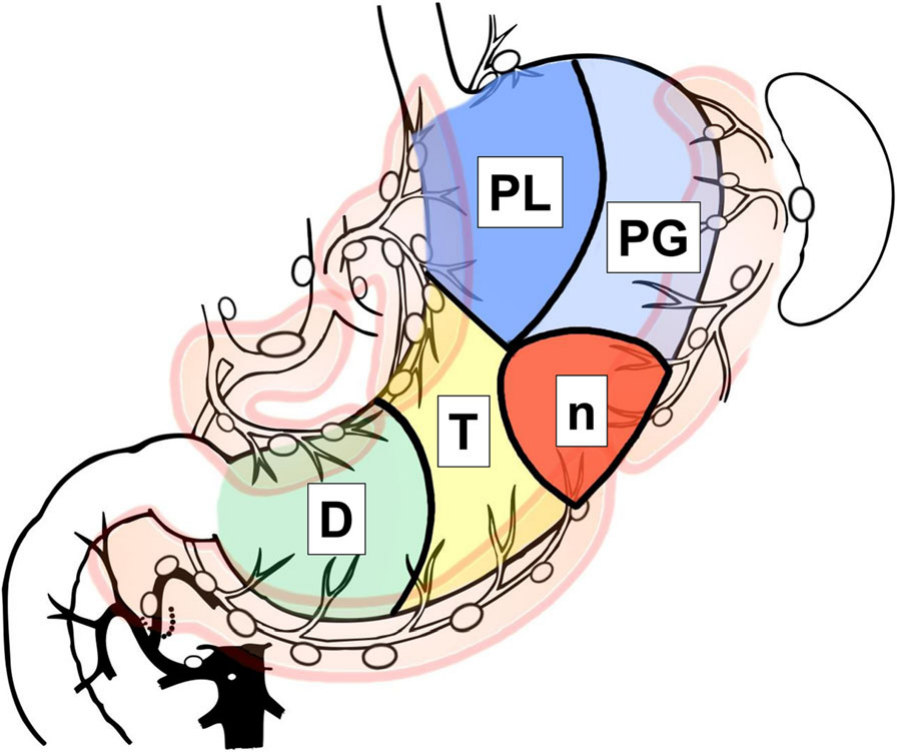
The nPTD classification. The ‘n’ zone is newly created as the region with a diameter of about 5 cm set on the watershed between the left and right gastroepiploic arteries on the greater curvature. The boundary between the P and T zones is changed to the line that links the watershed point between the left and right gastroepiploic arteries to the upper one-third point of the lesser curvature. Furthermore, the P zone is divided into two; the lesser curvature side (PL) and the greater curvature side (PG). The D zone is left unchanged. The lymphatic compartments are also displayed. This figure was newly created by the first author

The nPTD classification was developed by revising the PTD classification with these findings, as shown in Fig. 2. The boundary line changed because of challenging preoperative diagnosis of the boundary between the P and T zones. The point of greater curvature is the same as the watershed of the left and right gastroepiploic artery in the greater curvature. In contrast, to facilitate site determination preoperatively, the boundary point of the lesser curvature between U and M was changed to the upper one-third point because it is challenging to set the boundary of the old P and T zones preoperatively. In addition, a new circular region with a diameter of about 5 cm is set on the watershed on the greater curvature between the P and T zone as the ‘n’ zone. Furthermore, the P zone was divided into two sides: the lesser curvature (PL) and the greater curvature sides (PG). The D zone remained unchanged.

The nPTD classification reclassified the tumors, and lymphatic flow was examined, as shown in Table 6. In the PL zone, not only *r*-GA and *r*-GEA but also lymphatic flow to *l*-GEA was not observed. Moreover, there were no cases of lymphatic flow to the *l*-GEA in the new T-zone. Since the PL zone is set, there was no disadvantage to the patients in changing the boundary line of the lesser curvature of P and T zones. In contrast, cancer in the ‘n’ zone had extensive lymphatic flow into the three basins of *l*-GA, *l*-GEA, and *r*-GEA.

**Table 6:** The number and distribution of observed lymphatic compartments by nPTD classification

Tumor location	All site	PL	PG	n	T	D
Number of patients	416	48	15	16	154	183
Distribution of basin						
*l*-GA	83.2%	100.0%	86.7%	**100.0%**	89.6%	71.6%
*r*-GA	27.9%	**0.0%**	**0.0%**	0.0%	**0.0%**	63.4%
*r*-GEA	63.0%	**0.0%**	**0.0%**	**75.0%**	72.1%	76.0%
*l*-GEA	5.5%	**0.0%**	66.7%	**81.3%**	**0.0%**	0.0%
*p*-GA	1.4%	8.3%	13.3%	0.0%	0.0%	0.0%
#2	3.1%	16.7%	33.3%	0.0%	0.0%	0.0%
Number of basins						
1	33.9%	79.2%	20.0%	0.0%	38.3%	22.4%
2	48.3%	16.7%	60.0%	43.8%	61.7%	44.8%
3	17.8%	4.2%	20.0%	56.3%	0.0%	32.8%

All percentage numbers represent the percentage of confirmed lymphatic flow per tumor location

*#2* No. 2 lymph node, *l-GA* left gastric artery basin, *l-GEA* left gastroepiploic artery basin, *p-GA* posterior gastric artery basin, *r-GA* right gastric artery basin, *r-GEA* right gastroepiploic artery basin

## Discussion

The study aimed to update the PTD classification, especially to review the P and T zones. When the PTD classification was first published in 2008, the regional lymph nodes and D-number were classified based on the UML classification at that time [11]. Subsequently, the guidelines were revised; however, lymphatic flow was not emphasized in the current Japanese guideline.

Lymph node dissection must be based on lymphatic flow. Thus, it is necessary to focus on lymph flow to reduce the extent of lymph node dissection from D1+ without compromising curability. The sentinel node is a node that directly receives lymphatic drainage from a primary tumor [12]. The results of a multicenter prospective study showed that the sentinel node concept is valid for early gastric cancer [6]. In intraoperative node-negative cases diagnosed through sentinel node biopsy, it is thought that minimal nodal dissection limited to the lymphatic basin can be performed without compromising curability [5, 9], and a large-scale clinical trial to verify this is currently underway [12]. In addition, ICG fluorescence mapping has shown good results as an alternative to combination mapping [9]. In the present study, the size of the lesions was limited to ≤5 cm on the long axis. It was difficult to omit dissection due to the widespread lymphatic flow in gastric cancer > 5 cm, and there was a concern that the dye method could not accurately cover the lymphatic flow in large lesions.

The limitation of the old PTD classification is its difficulty in identifying the P and T boundary preoperatively. Intraoperatively, it is possible to determine this from the distribution of gastroepiploic arteries; however, it is challenging to determine the location of the tumor since the tumor is inside the stomach.

We observed that tumors located in watersheds of the greater curvature have extensive lymphatic flow into the three basins of *l*-GA, *l*-GEA, and *r*-GEA, and limited surgery should be cautiously performed on them. Thus, the ‘n’ zone was newly defined. The center of the ‘n zone’ is the watershed of the left and right gastroepiploic arteries in the greater curvature. This watershed corresponds to the constriction of the greater curvature of the body; therefore, it is easy to determine using the barium meal study. It is sometimes difficult to make a judgment with a gastrointestinal endoscopy, but it is a region that looks like a horse’s saddle when observed from the esophagogastric junction under sufficient inflation of air. The ‘n’ zone coincides with pre-linitis plastic cancer’s predominant site [13]. In addition, the boundary point in the lesser curvature was changed to the upper one-third point following the settings of the U and M boundary. This was a change in clarity. Furthermore, tumors confined to the P zone’s lesser curvature had no lymphatic flow to *l*-GEA, and the P zone was divided into two. If the tumor is localized to the PL, lymph node dissection of the *l*-GEA may be omitted. It is unclear why PL does not show lymphatic flow to the greater curvature, unlike T or D, but this is probably because the fornix has a large volume for receptive relaxation and the stomach wall is thin, resulting in less lymphatic confluence than that of the body or antrum.

**Fig. 3:**
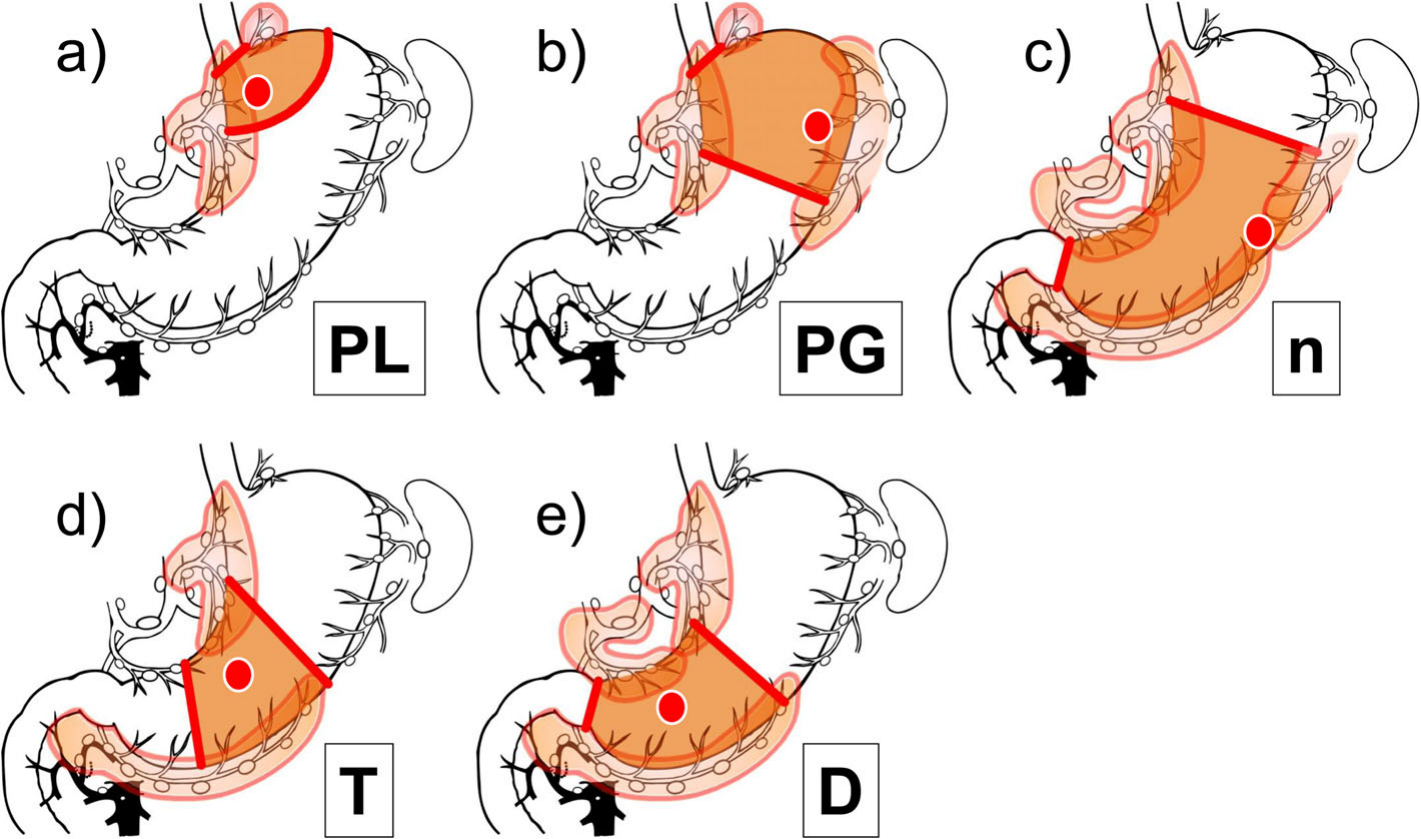
The recommendable surgical procedure for cT1N0 gastric cancer patients by nPTD classification. **a** Tumor in the PL zone. Mini-proximal gastrectomy and nodal dissection of *l*-GA, *p*-GA, and #2. **b** Tumor in the PG zone. Proximal gastrectomy and nodal dissection of *l*-GA, *p*-GA, #2, and *l*-GEA. **c** Tumor in the ‘n’ zone. Nearly-total gastrectomy (subtotal gastrectomy) and nodal dissection of D1+. **d** Tumor in the T zone. Segmental gastrectomy with long antral cuff and nodal dissection of *l*-GA and *r*-GEA. **e** Tumor in the D zone. Distal half gastrectomy and nodal dissection of *l*-GA, *r*-GA, and *r*-GEA. *l*-GA, left gastric artery basin; *r*-GA, right gastric artery basin; *r*-GEA, right gastroepiploic artery basin; *l*-GEA, left gastroepiploic artery basin; *p*-GA, posterior gastric artery basin. This figure is newly created by the first author

Based on these results, the appropriate resection and dissection range for cT1N0 patients according to the nPTD classification is shown in Fig. 3. Although sentinel node biopsy is required to diagnose node-negative cases since metastasis rarely spreads out of the basin in cT1N0 patients, the extent of nodal dissection can be limited to the lymphatic basins and dissection out of the basin is omitted without compromising cure [5–9]. For tumors located in D, distal half gastrectomy with nodal dissection of *l*-GA, *r*-GA, and *r*-GEA would be appropriate. In addition, segmental gastrectomy with nodal dissection of *l*-GA and *r*-GEA would be sufficient for tumors located in T [14–16]. If the tumor is located in the PG, a proximal gastrectomy would be considered appropriate [17]. However, if the tumor is localized within the PL, dissection of *l*-GEA can be omitted so that the resection area can be further reduced [18]. The reduction in the extent of resection area in proximal gastrectomy is of great significance to patients because it has been reported that a larger residual distal stomach is associated with less post-gastrectomy complaints [17]. As for curability, cancer recurrence after function-preserving gastrectomy is unlikely in patients diagnosed as node-negative pathologically. The problem in curability is when pathological nodal metastases are found postoperatively. Nevertheless, the lymphatic basin dissection is performed as a backup dissection; therefore, it is unlikely that all such patients will require additional dissection. The need for additional nodal dissection should be determined by considering the status of nodal metastases. However, despite standard lymph node dissection, five of 42 cases of lymph node metastasis in the present study had a recurrence. Adjuvant chemotherapy may be more useful than additional dissection. Conversely, for cancers located in the ‘n’ zone, nodal dissection of *l*-GA, *l*-GEA, and *r*-GEA is required, and, as a result, nearly-total gastrectomy is required. However, cT1N0 occupying the ‘n’ zone was rare, with only 3.8% of occurrence. Indeed, for gastric cancer deeper than cT2 and gastric cancer with cN+, either distal or total gastrectomy with D2 is usually required. In addition, even with cT1N0 tumors, lesions spanning more than one zone require both resection and dissection in a range that combines the extent of each resection and dissection area.

There were some limitations to this study. Since the nPTD classification was decided retrospectively, a prospective study will be necessary to validate this proposal. The optimal surgery for cT1N0 patients based on the nPTD classification may also be verified prospectively. The handling of the No. 9 lymph node (#9) is also unclear from our data. The frequency of metastasis in cT1N0 patients to #9 is reported to be low, and the therapeutic index is also low [19]. Although we excluded #9 from the node to be dissected in our study, it would be no problem to define #9 as the node to be dissected in all cases.

## Conclusions

Based on this in vivo observation of lymphatic flow, we developed the nPTD classification. Sentinel node biopsy for early gastric cancer is very useful for distinguishing node-negative cases and applying local resection; however, it requires several resources, and there are still unsolved issues [18]. Until these issues, such as rapid intraoperative diagnosis and adequate surgical procedures, are resolved, the nPTD classification will play an important role in performing the function-preserving gastrectomy as an alternative to the sentinel node biopsy.

## Data Availability

All data generated or analyzed during this study are included in this published article.

## Abbreviations

PTD: Proximal – Transitional – Distal
UML: Upper – Middle – Lower
RI: Radioisotope
ICG: Indocyanine green
PDE: Photodynamic eye
*l*-GA: Left gastric artery basin
*r*-GA: Right gastric artery basin
*l*-GEA: Left gastroepiploic artery basin
*r*-GEA: Right gastroepiploic artery basin
*p*-GA: Posterior gastric artery basin
